# Prognostic factors of resected pathological stage I lung adenocarcinoma: evaluating subtypes and PD-L1/CD155 expression

**DOI:** 10.1038/s41598-023-47888-x

**Published:** 2023-12-07

**Authors:** Natsumasa Nishizawa, Shohei Shimajiri, Rintaro Oyama, Takehiko Manabe, Yukiko Nemoto, Hiroki Matsumiya, Yohei Honda, Akihiro Taira, Masaru Takenaka, Koji Kuroda, Fumihiro Tanaka

**Affiliations:** 1 Second Department of Surgery, University of Occupational and Environmental Health, Japan, 1-1, Iseigaoka, Yahatanishiku, Kitakyushu, Fukuoka 807-8555 Japan; 2 Second Department of Pathology, University of Occupational and Environmental Health, Japan, 1-1, Iseigaoka, Yahatanishiku, Kitakyushu, Fukuoka 807-8555 Japan

**Keywords:** Lung cancer, Tumour biomarkers, Tumour immunology

## Abstract

We aimed to compare the prognostic impacts of adenocarcinoma subtypes, programmed death-ligand I (PD-L1), and CD155 expression on patients with resected pathological stage (p-stage) I lung adenocarcinoma. In total, 353 patients with completely resected p-stage I lung adenocarcinomas were retrospectively reviewed. The expression levels of PD-L1 and CD155 in tumour cells from each adenocarcinoma subtype were evaluated using several clinicopathological and histological features, such as the presence of a micropapillary pattern. A total of 52 patients (14.7%) had PD-L1-positive tumours, whereas 128 patients (36.3%) had CD155-positive tumours, with a tumour proportion score of 5% for both PD-L1 and CD155 expression. Compared with patients with other adenocarcinoma subtypes, those with solid-predominant adenocarcinomas were significantly more positive for PD-L1 and CD155. Multivariate analysis showed that PD-L1 expression status was significantly associated with progression-free survival and overall survival, whereas CD155 expression and the presence of a micropapillary pattern were not significantly associated with either parameter. Patients with PD-L1-positive tumours had poorer prognoses than those with CD155-positive tumours. Moreover, PD-L1 and CD155 were significantly expressed in solid-predominant adenocarcinomas. The results of this study suggest that immune checkpoint inhibitors can be used as adjuvants in the treatment of patients with p-stage I adenocarcinoma.

## Introduction

Lung cancer is a malignant disease with high morbidity and mortality rates worldwide. In Japan, it has the third highest morbidity and the highest mortality rate among all malignancies^1^. Non-small cell lung cancer (NSCLC) accounts for 80% to 90% of lung cancers, and patients with stage I NSCLC generally undergo surgery. However, the 5-year overall survival (OS) rates after complete resection of pathological stage (p-stage) I NSCLC reportedly range from 76.7 to 88.9%^2^. Several studies have explored the mechanisms underlying the recurrence and poor prognosis of NSCLC. These studies focused on driver gene mutations^3^, such as epidermal growth factor receptor^4^ and anaplastic lymphoma kinase^5^, and histological mechanisms, such as micropapillary pattern^6^ and tumour spread through air space^7, 8^. Recently, several immunological mechanisms have been elucidated.

Immune checkpoints are important in the cancer-immunity cycle because they regulate the immune balance between stimulatory and inhibitory mechanisms in the tumour microenvironment. Programmed cell death 1 (PD-1) is an immune checkpoint molecule expressed on activated cytotoxic T lymphocytes (CTLs). Cancer cells escape attack from CTLs by expressing PD-1 ligands, such as programmed death-ligand 1 (PD-L1), which inhibits the cytotoxicity of CTLs. Therefore, blockade of the PD-1/PD-L1 axis plays an important role in the modern systemic treatment of various malignant tumours, including lung cancer^9, 10^. The T-cell immunoglobulin and immunoreceptor tyrosine-based inhibitor domain (TIGIT) is also an immune inhibitory molecule expressed on CTLs. Cancer cells suppress T-lymphocyte activity by expressing TIGIT ligands, such as CD155, also known as poliovirus receptor (PVR)^11^. Accordingly, blockade of the TIGIT/CD155 axis has also emerged as a novel therapeutic strategy for various malignant tumours, including lung cancer^12–14^.

We previously reported the prognostic significance of PD-L1 and CD155 expression in 96 patients with completely resected pathological stage (p-stage) I lung adenocarcinoma between January 2003 and December 2006^15^. In the present study, we analysed immunohistological prognostic factors in a larger number of recent specimens than in our previous study. We aimed to evaluate the prognostic impacts of PD-L1 and CD155 expression and other previously unreported factors^15, 16^, such as adenocarcinoma subtypes, in a recent population with a similar background.

## Results

### Patient characteristics

A total of 353 patients were included in this study. All patients underwent lung resection via minimal thoracotomy. Sublobar resection was performed in 74 patients who did not undergo lobectomy (wedge resection in 37 patients and segmentectomy in 37 patients). Lymph node sampling was performed in 17 patients who underwent lobectomy or segmentectomy but were not fit for systemic nodal dissection. Meanwhile, 65 patients received adjuvant chemotherapy (carboplatin-based chemotherapy in 1 patient and tegafur uracil in 64 patients). The other patient characteristics are shown in Table 1.

**Table 1:** Patient characteristics.

		Total	Recurrence	Other	*p *value
	Patients	n = 353	n = 45	n = 308	
Age	Median	72	74	71	0.21
	Range	25–90	43–85	25–90	
Sex	Male	190	31	159	0.037
	Female	163	14	149	
Smoking history	Former/current	190	38	152	0.018
	Never	163	17	146	
P-stage	IA	235	21	214	0.004
	IB	118	24	94	
Tumor size (mm)	Median	21	25	20	0.0028
	Range	4–50	8–50	4–50	
Lymphovascular invasion	Positive	89	25	64	< 0.0001
	Negative	264	20	244	
Vascular invasion	Positive	61	15	46	0.005
	Negative	292	30	262	
Mode of lung resection	Lobectomy	279	33	246	0.33
	Sublobar resection	74	12	62	
Adenocarcinoma subtype	Lepidic	83	3	80	0.003
	Papillary	194	32	162	
	Achinar	26	3	23	
	Solid	25	4	21	
	Micropapillary	6	0	6	
	Invasive mucinous	17	2	15	
	Other	2	1	1	
	Micropapillary ≥ 5%	28	5	23	
PD-L1 TPS	≥ 5%	52	21	31	<0.0001
	< 5%	301	24	277	
CD155 TPS	≥ 5%	128	22	106	0.068
	< 5%	225	23	202	

### Histopathological features

The distributions of all adenocarcinoma subtypes and PD-L1/CD155 tumour proportion score (TPS) in all cases are shown in Table 2. In total, 52 patients (14.7%) had PD-L1-positive tumours, whereas 128 patients (36.3%) had CD155-positive tumours. Although more patients had predominantly lepidic or papillary adenocarcinoma, the patients with solid-predominant adenocarcinoma were significantly more positive for PD-L1 and CD155 than those with the other adenocarcinoma subtypes (both *p* < 0.0001) based on a TPS of 5% or higher in a previous study^15^.

**Table 2:** Adenocarcinoma subtypes.

Predominant subtypes	Patients	PD-L1 tumour proportion score			CD155 tumour proportion score		
		≥ 1%	≥ 5%	≥ 50%	≥ 1%	≥ 5%	≥ 50%
Lepidic	83	6 (7.2%)	2 (2.4%)	0 (0%)	24 (28.9%)	17 (20.5%)	8 (9.6%)
Papillary	194	43 (22.2%)	26 (13.4%)	3 (1.5%)	95 (49.0%)	69 (35.6%)	23 (11.9%)
Acinar	26	13 (50.0%)	7 (26.9%)	4 (15.4%)	15 (57.7%)	12 (46.2%)	5 (19.2%)
Solid	25	18 (72.0%)	15 (60.0%)	8 (32.0%)	20 (80.0%)	19 (76.0%)	14 (56.0%)
Micropapillary	6	4 (66.7%)	2 (33.3%)	0 (0%)	4 (66.7%)	4 (66.7%)	3 (50.0%)
Invasive mucinous	17	1 (5.9%)	0 (0%)	0 (0%)	7 (41.2%)	5 (29.4%)	2 (11.8%)
Other	2	1 (50/0%)	0 (0%)	0 (0%)	2 (100%)	2 (100%)	1 (50.0%)
Total	353	86 (24.4%)	52 (14.7%)	15 (4.2%)	167 (47.3%)	128 (36.3%)	56 (15.9%)

### Prognosis

The median follow-up duration after surgery was 1981 days. Two patients (both negative for PD-L1 and CD155) were lost to follow-up within five years of surgery.

**Figure 1:**
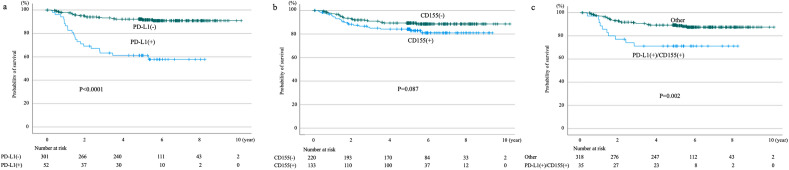
Progression-free survival curves according to PD-L1 expression status (**a**), CD155 expression status (**b**), and positive for both PD-L1/CD155 versus others (**c**).

**Figure 2:**
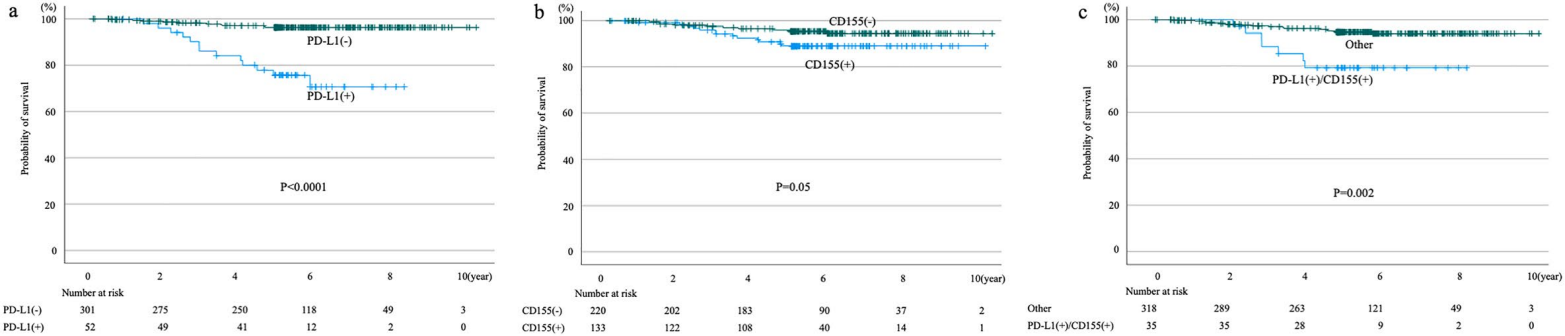
Overall survival curves according to PD-L1 expression status (**a**), CD155 expression status (**b**), and both PD-L1/CD155 positive versus others (**c**).

The 5-year progression-free survival (PFS) rates of the PD-L1-negative, PD-L1-positive, CD155-negative, and CD155-positive patients were 92.2%, 61.3%, 89.4%, and 84.2%, respectively. Significant differences in PFS rates were found between PD-L1-negative and -positive patients (*p* < 0.0001), but not between CD155-negative and -positive patients (*p* = 0.087). Meanwhile, significant differences in the 5-year PFS rates were noted between patients positive for both PD-L1 and CD155 and those positive for other markers (71.2% and 89.3%, *p* = 0.002; Fig. 1). Similarly, the 5-year cancer-specific overall survival (OS) rates of PD-L1-negative, PD-L1-positive, CD155-negative, and CD155-positive patients were 96.3%, 75.3%, 95.5%, and 89.1%, respectively. Significant differences in OS rates were found between PD-L1-negative and -positive patients (*p* < 0.0001), but not between CD155-negative and -positive patients (*p* = 0.05). Meanwhile, significant differences in the 5-year OS rates were noted between patients positive for both PD-L1 and CD155 and those positive for other markers (79.3% and 94.7%, respectively; *p* = 0.002; Fig. 2).

Multivariate analysis showed that PD-L1 expression status (hazard ratio [HR] = 4.61, 95% confidence interval [CI] = 2.30–9.22, *p* < 0.001, q < 0.001) was significantly associated with PFS. Moreover, PD-L1 expression status (HR = 5.46, 95% CI = 2.11–14.1, *p* < 0.001, q = 0.0018) and mode of lung resection (HR = 4.49, 95% CI = 1.77–11.4, *p* = 0.002, q = 0.007) were significantly associated with OS. CD155 expression and the presence of a micropapillary pattern were not significantly associated with PFS or OS. PD-L1 expression status was the factor most significantly associated with PFS and OS (Tables 3 and 4).

**Table 3:** Hazard ratio for progression-free survival.

Covariates	Univariate			Multivariate			
	Hazard ratio	95% confidence interval	*p *value	Hazard ratio	95% confidence interval	*p *value	*q*-value
Age (continuous)	1.02	0.99–1.06	0.17				
Sex (male vs. female)	2.09	1.11–3.92	0.022	0.63	0.33–1.19	0.16	0.24
Smoking (former/current vs. never)	1.52	0.83–2.79	0.17				
Pathological stage (IA vs. IB)	2.44	1.36–4.39	0.003	2.09	1.12–3.88	0.02	0.06
Vascular or Lymphovascular invasion (positive vs. negative)	3.66	2.02–6.62	< 0.001	1.78	0.90–3.53	0.10	0.20
Mode of lung resection (sublobar resection vs. lobectomy)	1.59	0.82–3.09	0.17				
Adjuvant chemotherapy (performed vs. not performed)	0.60	0.32–1.13	0.12				
Micropapillary pattern (≥ 5% vs. others)	1.64	0.65–4.16	0.29	1.08	0.42–2.76	0.87	0.87
PD-L1 expression status (TPS ≥ 5% vs. others)	5.91	3.29–10.6	< 0.001	4.61	2.30–9.22	< 0.001	< 0.001
CD155 expression status (TPS ≥ 5% vs. others)	1.65	0.92–2.97	0.09	0.77	0.40–1.48	0.44	0.53

**Table 4:** Hazard ratio for overall survival.

Covariates	Univariate			Multivariate			
	Hazard ratio	95% confidence interval	*p* value	Hazard ratio	95% confidence interval	*p* value	*q*-value
Age (continuous)	1.00	0.96–1.04	0.99				
Sex (male vs. female)	3.48	1.29–9.35	0.014	0.37	0.13–1.04	0.06	0.11
Smoking (former/current vs. never)	2.13	0.88–5.17	0.96				
Pathological stage (IA vs. IB)	1.92	0.85–4.35	0.12	2.49	0.99–6.28	0.052	0.12
Vascular or Lymphovascular invasion (positive vs. negative)	4.00	1.73–9.25	0.001	2.11	0.81–5.47	0.13	0.18
Mode of lung resection (sublobar resection vs. lobectomy)	3.27	1.41–7.51	0.006	4.49	1.77–11.4	0.002	0.007
Adjuvant chemotherapy (performed vs. not performed)	0.66	0.27–1.62	0.37				
Micropapillary pattern (≥ 5% vs. others)	1.74	0.23–12.8	0.59	0.46	0.06–3.49	0.45	0.53
PD-L1 expression status (TPS ≥ 5% vs. others)	7.88	3.45–17.9	< 0.001	5.46	2.11–14.1	< 0.001	0.0018
CD155 expression status (TPS ≥ 5% vs. others)	2.22	0.98–5.07	0.06	0.86	0.34–2.14	0.74	0.74

## Discussion

In the present study, the distribution of PD-L1/CD155 and subtypes in p-stage I adenocarcinoma and their relationship with prognosis after complete lung resection were evaluated. The expression levels of PD-L1 and CD155 were significantly higher in solid-predominant adenocarcinomas than in the other subtypes. PD-L1 expression was a significant prognostic factor for poor OS and PFS, whereas CD155 expression had no significant prognostic value. This result suggests that the inhibition of the PD-1/PD-L1 axis is more important than that of the TIGIT/CD155 axis in treating p-stage I adenocarcinoma, especially solid-predominant adenocarcinoma.

Several studies have reported the clinical importance of PD-L1 expression in lung cancer; the relationship between the tumour PD-L1 expression status and prognosis^16, 17^, and the response to immune checkpoint inhibitors such as anti-PD-1 antibodies (i.e. nivolumab^18^ and pembrolizumab^19^) and anti-PD-L1 antibodies (i.e. atezolizumab^20^ and durvalumab^21^). In addition, adjuvant therapy using immune checkpoint inhibitors has recently been reported. Felip et al.^22^ demonstrated that using atezolizumab as adjuvant therapy for patients with stage II to IIIA NSCLC improves the PFS rates of patients expressing PD-L1 in ≥ 1% of tumour cells (HR = 0.66, 95% CI = 0.50–0.88, *p* = 0.0039) compared with those of patients receiving the best supportive care (IMpower 010 study). Although we analysed only p-stage I adenocarcinoma in this study, the results may help encourage the use of immune checkpoint inhibitors as adjuvant therapy.

The relationship between CD155 and lung cancer has been reported in several studies. We previously reported that p-stage I adenocarcinoma expressing both CD155 and PD-L1 has a significantly poorer prognosis^15^. Moreover, NSCLC expressing both PD-L1 and TIGIT (ligand for CD155) has a poorer prognosis after neoadjuvant chemoradiotherapy^14^. Sun et al.^23^ reported that CD155 expression is an independent poor prognostic factor in 334 lung adenocarcinomas (including 137 p-stages II–IV). Lee et al.^24^ found that patients with lung squamous cell carcinoma expressing both CD155 and PD-L1 show poor prognosis. However, CD155 expression was not a significant prognostic factor for PFS or OS in the present study. As a related report to this result, using tiragolumab (anti-TIGIT antibody) plus atezolizumab for the treatment of advanced or metastatic NSCLC with strong PD-L1 expression does not meet its co-primary endpoint of PFS (phase III SKYSCRAPER-01 study)^25^. Thus, the expression of CD155 may not have much effect on prognosis in p-stage I lung adenocarcinoma (for reference, there was no significant difference in PD-L1 TPS between the CD155-positive and -negative groups: *p* = 0.85). CD155 overexpression causes tumour progression by promoting the migration and invasion of cancer cells and inducing immune escape^26^, and this mechanism is more pronounced in more advanced lung cancers. Therefore, the results may be different in more invasive histologies or advanced stages, and treatment of the CD155 axis may not contribute as much to an improved prognosis. A similar analysis should be planned for stage II or more advanced-stage adenocarcinomas or other histologies. Moreover, further studies on anti-TIGIT antibodies are warranted.

Focusing on adenocarcinoma subtypes in this study, we found the distribution of PD-L1 and CD155 expression in each subtype, and significant expression of both PD-L1 and CD155 in solid adenocarcinoma. Similarly, Miyazawa et al.^27, 28^ reported that PD-L1-positive tumours are more frequent in acinar- and solid-predominant adenocarcinomas than in other adenocarcinoma subtypes. The results of the present study suggest the clinical significance of examining tumour PD-L1 expression and using anti-PD-1 or anti-PD-L1 antibodies as adjuvant therapy for patients with solid-predominant adenocarcinoma, even at p-stage I. We could not find any reports describing the relationship between CD155 expression and adenocarcinoma subtype when searching MEDLINE. Therefore, this study is the first to report the distribution of CD155 expression in p-stage 1 adenocarcinoma subtypes. Several studies have reported that the micropapillary pattern in lung adenocarcinoma is associated with poor prognosis and lymphovascular invasion^29–31^. However, in the present study, the presence of a micropapillary pattern was not a significant prognostic factor for PFS or OS. Thus, similar to CD155, the presence of a micropapillary pattern possibly becomes a poor prognostic factor in more advanced stages of the disease.

The present study has several limitations. First, this was a retrospective single-centre study. Second, the present study provided no data on p-stage II–III disease. Finally, histological types other than adenocarcinomas were not analysed. Previous studies have shown that CD155 expression is significantly higher in advanced stages and other histological types, such as squamous cell carcinoma^23, 24^. A larger-scale multicentre study is warranted to reveal PD-L1 and CD155 expression, tumour subtypes, and their association with prognosis in other types of NSCLC or advanced-stage lung cancer.

In conclusion, PD-L1 expression status was associated with a poorer prognosis than CD155 expression status. Moreover, both PD-L1 and CD155 were significantly expressed in solid-predominant p-stage I adenocarcinoma. The results of this study may promote the use of immune checkpoint inhibitors as adjuvant therapies. Further research is warranted to provide novel insights into lung cancer and its immunohistological mechanisms.

## Materials and methods

### Patient selection

We retrospectively evaluated consecutive patients with p-stage I lung adenocarcinoma who underwent complete resection without any induction treatment for lung cancer at the Second Department of Surgery (Chest Surgery), University of Occupational and Environmental Health, Japan between January 2012 and December 2017. P stage was determined in accordance with the TNM classification (Union for International Cancer Control TNM staging system, 7th edition).

This study was approved by the Institutional Review Board of the University of Occupational and Environmental Health (approval no. H26 15; Kitakyushu, Japan) and performed in accordance with the Declaration of Helsinki. All the participants provided written informed consent. Patients who did not provide informed consent and those without sufficient tumour cells for immunohistochemistry (IHC) were deemed ineligible and excluded from the study. All methods were performed in accordance with relevant guidelines and regulations.

### Histopathological classification

**Figure 3:**
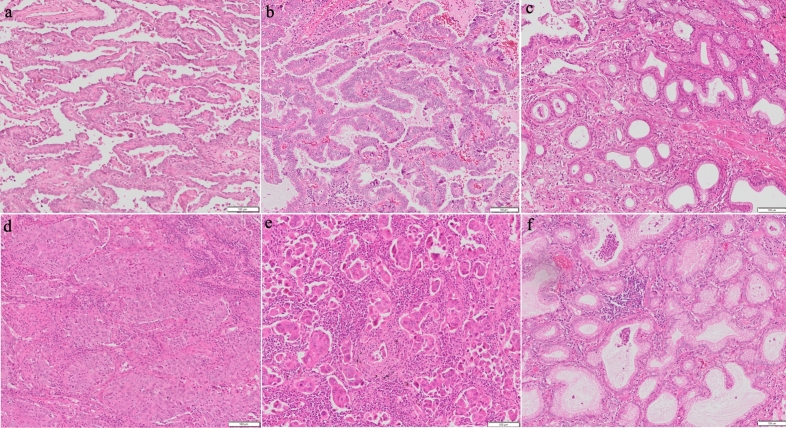
Haematoxylin and eosin stain of the adenocarcinoma subtypes, including (**a**) lepidic, (**b**) papillary, (**c**) acinar, (**d**) solid, (**e**), micropapillary, and (**f**) invasive mucinous.

For the diagnosis of adenocarcinoma subtypes, serial 4 μm sections were cut from each formalin-fixed and paraffin-embedded primary tumour specimen and stained with haematoxylin and eosin (HE). Each HE slide was observed and evaluated for tumour cell counts and subtypes (Fig. 3). The percentage of each subtype was determined, and the subtype with the highest percentage was considered for subsequent analyses.

### Immunohistochemistry

Sections with a sufficient number of tumour cells were incubated with an anti-PD-L1 monoclonal antibody (clone E1L3N; Cell Signaling Technology, Inc.) or an anti-CD155 monoclonal antibody (clone D8A5G; Cell Signaling Technology, Inc.) in accordance with the manufacturer’s protocol^32^. For antigen retrieval, sections for PD-L1 analysis were heated in 1 mmol/L ethylenediaminetetraacetic acid (pH 8.0) at 98 °C for 15 min. Similarly, sections for CD155 analysis were heated in 1X citrate unmasking solution (pH 6.0) at 98 °C for 10 min and then cooled on a bench top for 30 min. After antigen retrieval, the sections were incubated in 3% hydrogen peroxide for 10 min to inactivate endogenous peroxidase. After being blocked with Protein Block Serum-Free (Agilent Technologies, Carpinteria, CA) for 30 min, the sections were incubated with primary antibodies for each antigen diluted at 1:200 for 1 h at room temperature. They were then washed and incubated with SignalStain Boost IHC Detection Reagent HRP Rabbit (Cell Signaling Technology) for 30 min. Thereafter, the sections were visualised using DAB + liquid (Agilent Technologies, Santa Clara, CA, USA) and counterstained with haematoxylin.

**Figure 4:**
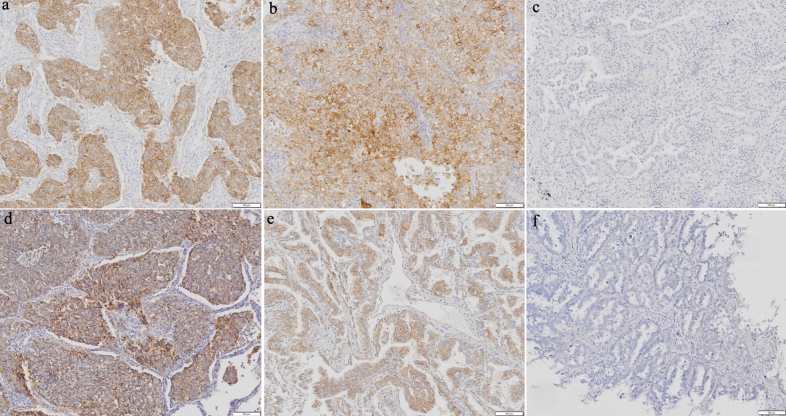
Immunohistochemical detection of PD-L1 (**a**, **b**: positive, **c**: negative) and CD155 (**d**, **e**: positive, **f**: negative) expression.

Each slide was examined independently by two investigators who were blinded to clinical data. In cases of disagreement between the two investigators, consensus was reached through simultaneous examination using a double-headed microscope. Each cancer cell line was considered positive if the membrane was stained with any intensity (Fig. 4).

One of the main purposes of the present study was to validate our previous study^15^. In the 96 patients included in our previous study^15^, PD-L1 expression (cut-off value, 5%) was found to be a marginal factor to predict a poor prognosis^16^. Receiver operating characteristic (ROC) curves were generated by plotting the false-positive rate of a model against its true positive rate for prediction of tumour recurrence, and the area under the curve was calculated to determine an optimal cut-off value of TPS for CD155. Results showed that the TPS value of 5% was the optimal cut-off value, with a sensitivity of 71.4% and a specificity of 67.1% (the area under the ROC curve: 0.677, 95% confidence interval: 0.531–0.823). Therefore, the optimal cut‑off value for classifying positive cells in the present study was determined using a 5% TPS for both CD155 and PD-L1 expression to align our resected sample evaluation methods.

### Patient follow-up

For each patient, a routine follow‑up was performed at the outpatient clinic as follows: chest roentgenography every 3 months; chest computed tomography, brain magnetic resonance imaging, and bone scan every 6 months for the first 3 years after surgery; all examinations were performed annually thereafter. Additional examinations were performed when symptoms or signs of recurrence were detected. Telephone follow‑up was performed if the patient did not come to our clinic for routine follow‑up.

### Statistical analysis

Categorical data were compared using Fisher’s exact test. Continuous data were compared using a non-parametric test (Mann–Whitney U test). The Kaplan–Meier method was used to estimate the probability of OS and PFS, and survival differences were analysed using the log‑rank test. Univariate and multivariate analyses were performed using a Cox proportional hazards regression model to identify independent prognostic factors. Significant prognostic factors in the univariate analysis and potentially poor prognostic factors, including sex, p-stage (IA or IB), PD-L1 and CD155 expression status, and the presence of a micropapillary pattern, were included as covariates in the multivariate analysis. In multivariate Cox proportional hazards regression analysis, q-values were calculated using the Benjamini–Hochberg method^33^ to adjust for multiple comparisons. False discovery rate was set at 0.05.

All statistical analyses were performed using SPSS version 27 software (IBM Corp., Armonk, NY, USA). Differences were considered statistically significant at *p* < 0.05 (and q < 0.05 in multivariate analysis).

### Ethics approval and consent to participate

This study was approved by the Institutional Review Board of the University of Occupational and Environmental Health (Approval no. H26‑15; Kitakyushu, Japan). All participants provided written informed consent to participate in the present study.

## Data Availability

The datasets used and/or analysed during the present study are available from the corresponding author upon reasonable request.
